# Hyaluronan-based hydrogels as dermal fillers: The biophysical properties that translate into a “volumetric” effect

**DOI:** 10.1371/journal.pone.0218287

**Published:** 2019-06-11

**Authors:** Annalisa La Gatta, Rosanna Salzillo, Claudia Catalano, Antonella D’Agostino, Anna Virginia Adriana Pirozzi, Mario De Rosa, Chiara Schiraldi

**Affiliations:** 1 Department of Experimental Medicine, School of Medicine, University of Campania “Luigi Vanvitelli”, Naples, Italy; 2 Centro Regionale di Competenza in Biotecnologie Industriali BioTekNet S.C.p.A. c/o University of Campania “Luigi Vanvitelli”, Naples, Italy; Brandeis University, UNITED STATES

## Abstract

Biophysical and biochemical data on hyaluronan (HA)-based dermal fillers strongly support their optimal use and design to meet specific requisites. Here, four commercially available (in Europe) HA “volumetric” fillers, among the most used in the clinical practice, have been characterized *in vitro*. Analyses revealed the highest amounts of water-soluble HA reported so far and provided hydrodynamic data for these soluble polymeric fractions. Volumetric gels exhibit a wide range of rigidity with most of them showing G’ values around 200-300Pa. They greatly differ in cohesivity. 1mL of gel hydrates up to 2.4–3.2mL. The products completely solubilize due to Bovine Testicular Hyaluronidase (BTH)’s action, thus predicting *in vivo* complete resorption. For the first time, filler degradation due to reactive oxygen species (ROS) was studied by rheological measurements and a rank in stability was established. Studies using Human Dermal Fibroblasts (HDF) indicated a positive biological response to the HA networks. Further, gel capacity to prompt collagen I, elastin and aquaporin3 synthesis was demonstrated, thus suggesting a positive effect on skin elasticity and hydration, besides the physical volumetric action. The findings are the first wide assessment of features for the volumetric class of HA-fillers and include first data on their resistance to degradation by ROS and biological effects on HDF. The study represents a valuable contribution to the understanding of HA-fillers, useful to optimize their use and manufacture.

## Introduction

Hyaluronan (HA)-based hydrogels, obtained by crosslinking the biopolymer with 1,4 butandiolediglycidylether (BDDE), are the most commonly used dermal fillers in aesthetic medicine procedures for facial rejuvenation [1–4]. Diverse proprietary manufacturing technologies (i.e., Vycross, NASHA, CMP etc.) have been developed and are currently employed for HA filler production [3,5,6]. As the understanding of processes governing facial aging improves, HA gel design is being upgraded to match specific clinical needs. As a consequence, each brand markets a line of formulations, possibly based on the same technology but tuned to address the diverse needs for a full face restoration.

At least one gel, in each line, is mainly indicated to restore volume loss from age-related bone and fat resorption. Injection into the deep dermis down to the pre-periosteal area is recommended for this type of product, that can be classified as “volumetric” fillers [7–10]. “Global action” and “skinbooster” fillers, intended to treat more superficial depressions, with the latter especially indicated to improve skin quality, are also released from each brand [11–13]. Diverse biophysical properties are expected to be at the basis of the above classification. For instance, highly deformable gels are generally suggested for superficial injections, while volume restoration is achieved by using more rigid products able to maintain their shape under the stress of facial movements. Biophysical features of the gels are in turn dependent on manufacturing parameters such as the biopolymer crosslinking extent, the final gel concentration, the amount of soluble polymer in the formulation etc. [14–21].

The literature is now in agreement on the importance of the biophysical characterization of HA fillers to guide physicians in selecting the most appropriate product, depth of injection and administration technique for an optimal clinical outcome [1, 3, 6, 22–27]. Additionally, such evaluations, combined with clinical studies, are key to tailor gels towards specific requisites. On this basis, here we aimed to ascertain the biophysical properties of a filler that translate into a volumetric effect. To accomplish this, four among the most popular available “volumetric” gels were studied in terms of composition (soluble/insoluble HA concentration), hydration capacity, rheological behavior (stiffness, elasticity, complex viscosity), cohesivity and resistance to Bovine Testicular Hyaluronidase (BTH)-catalyzed hydrolysis. In addition, a method to evaluate filler sensitivity to degradation by Reactive Oxygen Species (ROS) is presented. Finally, a biological experimentation using human dermal fibroblasts was performed to evaluate and compare the effect of the fillers on skin restoration.

The results were expected to provide a complete panel of properties for the volumetric gels potentially highlighting differences in behavior thus interestingly contributing to enhance HA-fillers knowledge and to optimize their use and manufacture.

## Materials and methods

### Materials

Restylane Lift (R_L_) is distributed by *Galderma S*.*P*.*A*. Juvederm Voluma (J_V_) with Lidocaine is distributed by *Allergan S*.*P*.*A*. (Pringy, France). Teosyal RHA4 (T_RH4_) is distributed by *Teoxane SA* (Geneva, Switzerland). Aliaxin SV (A_SV_) is distributed by *IBSA Farmaceutici* Italia srl (Milan, Italy).

They are HA-based dermal fillers intended for the use as volumetric gels [7–10]. They all consist in BDDE-crosslinked HA, suspended in physiological medium. The HA concentration values, as reported in the package inserts, are 20mg/mL for R_L_ and J_V,_ 23mg/mL for T_RH4_ and 25mg/mL for A_SV_ [7–10] R_L_, J_V_ and T_RH4_ also contain 0.3% lidocaine.

Bovine testicular hyaluronidase, BTH (EC 3.2.1.35), salt-free lyophilized powder with a specific activity of 1275U/mg was purchased from *Sigma-Aldrich S*.*R*.*L*. (Milan, Italy) (cat. N. H3884, lot. SLBF8562V).

Dulbecco’s Phosphate Buffered Saline (PBS) without calcium and magnesium was purchased from *Lonza Sales Ltd*, Switzerland (cat. N. BE17-516F, lot. N. 3MB191).

Hydrogen peroxide, 30% w/w in water was purchased from Sigma Aldrich, cat. N.H1009. Copper (II) sulfate (≥99%) was purchased from Fluka, cat. N. 61230.

### Biophysical characterization

#### Soluble fraction quantification and hydrodynamic characterization

The water-soluble fraction of the gels was quantified as reported elsewhere [18, 21]. Briefly, the gels were diluted to 4 mg/mL in PBS (1.0mL final volume). The suspensions obtained were continuously stirred (1000rpm) for 24h at 37°C, then centrifuged (13000 × g, 5 min). The supernatant was filtered through 0.22μm. The HA content (mg/mL) in the filtered samples (water-soluble HA fraction) was quantified by the carbazole assay [21, 28]. Based on the total HA concentration indicated in the package inserts, the amount of water-insoluble HA (mg/mL) in each gel was calculated.

The hydrodynamic parameters for the soluble fraction of the gels were determined by the Size Exclusion Chromatography-Triple Detector Array (SEC-TDA) equipment by Viscotek (*Viscotek*, *Malvern*, *UK*).

A detailed description of the SEC-TDA system, of its potential to analyse biopolymers such as HA, and of the analysis conditions are reported elsewhere [21, 29, 30]. Sample’s molecular weight (M_w_, M_n_, M_w_/M_n_), molecular size (hydrodynamic radius-R_h_) and intrinsic viscosity ([η]) distributions were derived. The Mark–Houwink–Sakurada (MHS) curve (log[η] vs logM_w_) was directly derived for each sample.

#### Hydration capacity

The hydration extent of the fillers was determined as previously reported, with slight modifications [18, 21]. Specifically, 0,1mL (equivalent to 0,1g) of each filler was incubated in PBS (1.0mL final volume) at 37°C under stirring (1000rpm) for 16h. After centrifugation (13000 × g, 5 min) and supernatant removal, the pellet was weighed obtaining the hydrated sample mass (g), corresponding to the hydrated sample volume (mL) (density equal to 1g/mL). The hydration extent of each gel was calculated as:

$$gel\prime s\;hydration\;capacity\;(\frac{mL}{mL})=\frac{hydrated\;sample\;volume\;(mL)}{initial\;sample\;volume\;(mL)}$$ (1)

Such values represent the volume expansion for each formulation, when allowed to reach the equilibrium swelling in PBS.

The hydration capacity referred to the water-insoluble HA in each formulation (mg) was also calculated as:

$$insoluble\;HA^{\prime }s\;hydration\;capacity\;\left(\frac{mL}{mg\;of\;{HA}_{ins}}\right)=\frac{gel\;volume\;at\;equilibrium\;(mL)}{insoluble\;HA\;in\;the\;gel\;(mg)}$$ (2)

#### Rheological characterization

The rheological behaviour of the gels was investigated as previously reported for similar formulations [21, 31]. Briefly, a Physica MCR301 oscillatory rheometer (*Anton Paar*, Germany), equipped with a parallel plate geometry, 25 mm plate diameter, 1.0 mm gap and a Peltier temperature control, was used. Measurements were performed at 37°C. The dynamic moduli and the complex viscosity of the gels were measured as a function of the oscillation frequency (0.159 -10Hz) at 0.1% strain. Analyses were performed in duplicate. Representative curves are reported.

#### Stability to degradation

Gels were evaluated for their sensitivity to degradation due to ROS- and BTH-action. Stability of the gels to ROS action was studied using the H_2_O_2_/Cu^2+^ system for generating radicals [32]. Aqueous solutions of H_2_O_2_ and CuSO_4_ were added to 1mL of each gel to have H_2_O_2_ 375mM and CuSO_4_ 3.75mM (1.3ml final volume). The suspensions were mixed and rapidly placed on the lower plate of the rheometer. A time oscillatory test was carried out at 37°C. Specifically, the storage modulus value of the samples was measured as a function of the time while maintaining constant the frequency and the strain. The delay between the addition of the H_2_O_2_/Cu^2+^ system to the samples and the acquisition of the first G’ value was 5 minutes. Great attention was paid to create the same testing conditions for all the analyzed samples (mixing time, time for equilibrating on the rheometer’s plate etc.). For each gel, the same experiment was performed adding water in place of the H_2_O_2_/Cu^2+^ system (control). Degradation was monitored by measuring the G’ decrease (% in respect to the control) as a function of the incubation time (up to 15’) with the ROS generating system. Analyses were performed in duplicate. Results are reported as the mean value ± SD.

Sensitivity of the gels to enzymatic degradation was evaluated by measuring the increase in the water-soluble HA fraction amount of fillers due to BTH action [18, 21, 31]. Briefly, gels diluted to 4 mg/mL in PBS were incubated for 4h in the presence of BTH or 100 U/mL) at 37°C under stirring. The suspensions were then withdrawn and boiled for 10 min to inactivate the enzyme. The samples were filtered on 0.22 μm. The water-soluble HA fraction (mg/mL) was quantified by the carbazole assay as reported in the previous paragraph (2.2.1).

#### Cohesivity

Product cohesivity was evaluated following the validated protocol reported by Sundaram and collaborators [33]. Specifically, 1g of the gel was homogeneously stained by adding 10 μl of toluidine blue (0.1% w/w in phosphate buffer, pH7.4) Samples were carefully drawn into 1mL syringes and extruded (without the needle), under reported conditions, in 1L beaker containing water and equipped with a magnetic stirrer [33]. Immediately after extrusion, the magnetic stirring and filming started; in addition, images were obtained at specific times (15, 70 and 90seconds).

Cohesivity was evaluated independently by 4 raters that assigned, for each sample, at each time, a value of cohesivity (from 1 to 5) referring to the Gavard-Sundaram Cohesivity Scale [33]. Results were reported as the mean score ± SD.

### Biological evaluation

#### Cell cultures

A human dermal fibroblast cell line immortalized with hTERT (HDF cells, BJ-5ta, ATCC CRL-4001) was cultured in a 4:1 mixture of DMEM and Medium199 supplemented with 0.01mg/ml hygromycin B and 10% (v/v) FBS. All materials for HDF culture were purchased from ATCC (USA). The cells were grown on tissue culture plates (BD Falcon, Italy), using an incubator with a humidified atmosphere (95% air/5%CO_2_ v/v) at 37°C.

#### Cell viability (MTT test)

Cells were seeded at a density equal to 2 x10^4^ cells /cm^2^. 24h after seeding, cells were treated with fillers. Specifically, the gels were added to the medium at 0.16% w/w final concentration. After 24 and 48h of treatments, the medium was removed and after exhaustive washing in physiological solution, cells were observed at inverted optical microscope. Then, cell viability was assessed by measuring the reduction of the tetrazolium dye 3-(4,5-dimethylthiazol-2-yl)-5-(3 carboxymethoxyphenyl)-2-(4- sulfophenyl)-2H–tetrazolium (MTT). Cell viability in presence of fillers was reported in respect to untreated cells (% viability) [34].

#### Western blotting for Collagen type 1, Elastin, Aquaporine 3 and Actin

After 48h of treatment with the gels, HDF cells were washed three times with physiological solution. Cells were then lysed in RIPA buffer (Cell Signaling Technology) and proteins were extracted. The protein concentration was determined using Bradford method with Bio-Rad protein assay reagent (Bio-Rad Laboratories, Milan, Italy). Equal amounts of protein (30 μg) were loaded on 12% SDS-PAGE gels. A western blotting analysis was carried out as previously reported [35,36]. The separated proteins were then transferred to nitrocellulose membrane (Amersham). The membrane was blocked in 5% w/v milk dissolved in Tris-buffered saline and 0.05% Tween-20. Collagen type 1 (180KDa), Elastin (60kDa), Actin (48KDa) and Aquaporine (25KDa) primary antibodies were used at 1:200, 1:250, 1.1000 and 1:250 v/v dilutions respectively at room temperature for 2 h (all antibodies purchased from Santa Cruz Biotechnology, CA, USA). Membranes were then washed three times for 10 min in TBS-Tween. Immunoreactive bands were detected by chemiluminescence using corresponding horseradish peroxidase-conjugated secondary antibody (Santacruz Biotechnology; 1:5000 dilutions) incubated with a 1: 10000 dilution for 1 h. Blots were developed using the ECL system (Chemicon-Millipore) according to the manufacturer’s protocol. Protein levels were normalized with respect to the signal of anti actin polyclonal antibody using as housekeeping protein (Santacruz Biotechnology; 1:1000 dilutions). The semi-quantitative analysis of protein levels was carried out by the Gel Doc 2000 UV System and the Gel Doc EZ Imager (Quantity one software, Bio-Rad Laboratories).

### Data analysis

Unless otherwise indicated, experiments were performed at least in triplicate, results were reported as the mean value ± SD and the data were statistically evaluated using SigmaPlot 14. One-way ANOVA tests followed by post hoc tests (with Holm-Sidak correction for multiple comparison) were run. The level of significance was fixed at 0.05.

## Results

### Water-soluble fraction of fillers: Quantitative determination and hydrodynamic analysis

**Fig 1:**
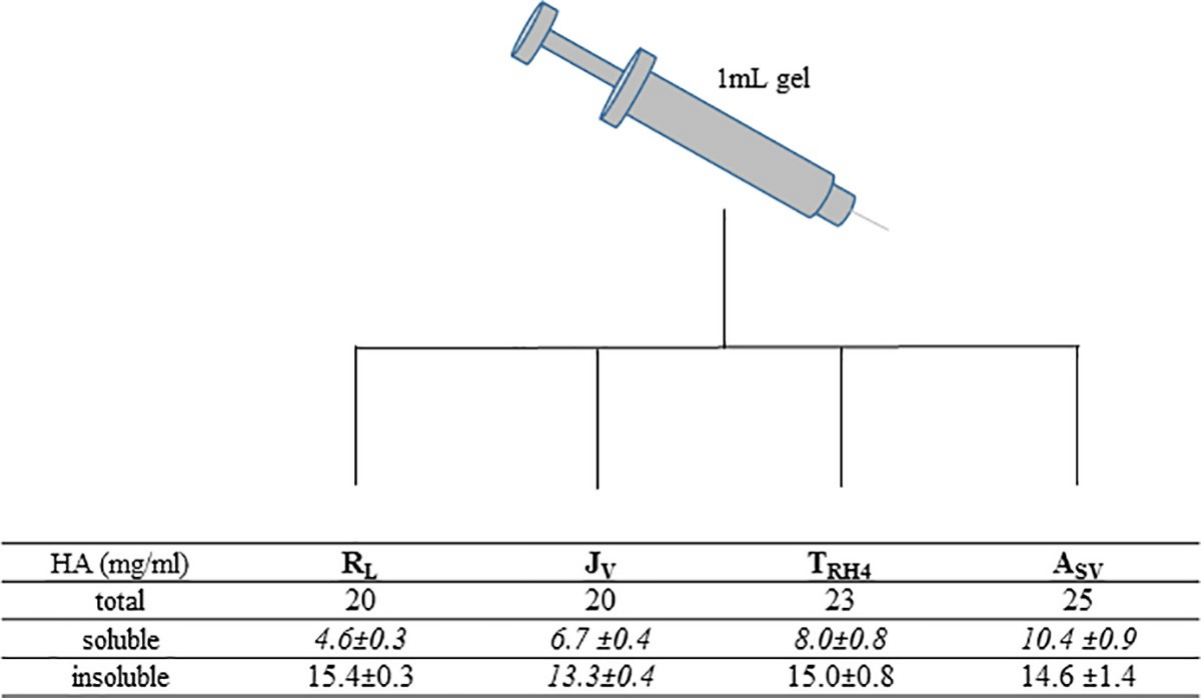
Results of the gel composition analyses. The total HA concentration in the gels, as indicated in the package inserts, is shown in the first row. The amount of water-soluble HA in each gel (mg/mL), based on our analyses, is then reported. In *Italics*, values significantly different (p<0.05). The amount of water-insoluble HA, derived from the previous data, is finally indicated. In *Italics*, values significantly different (p<0.05).

The results from gel composition analyses are reported in Fig 1. The analyses revealed water-soluble HA in all the gels tested. Specifically, the water-soluble HA amount ranged from 4.6 (R_L_) to about 10.4mg/mL (A_Sv_). A_SV_, the most concentrated in total HA, was also the most concentrated in soluble HA, followed by T_RH4_ and, then, by J_V_ while R_L_ is the gel with the lower water-soluble HA content.

J_V_ proved the less concentrated in water-insoluble HA, while even if differing for the total HA concentration, A_SV_, R_L_ and T_RH4_ were comparable for the insoluble HA amount (p>0.05).

**Fig 2:**
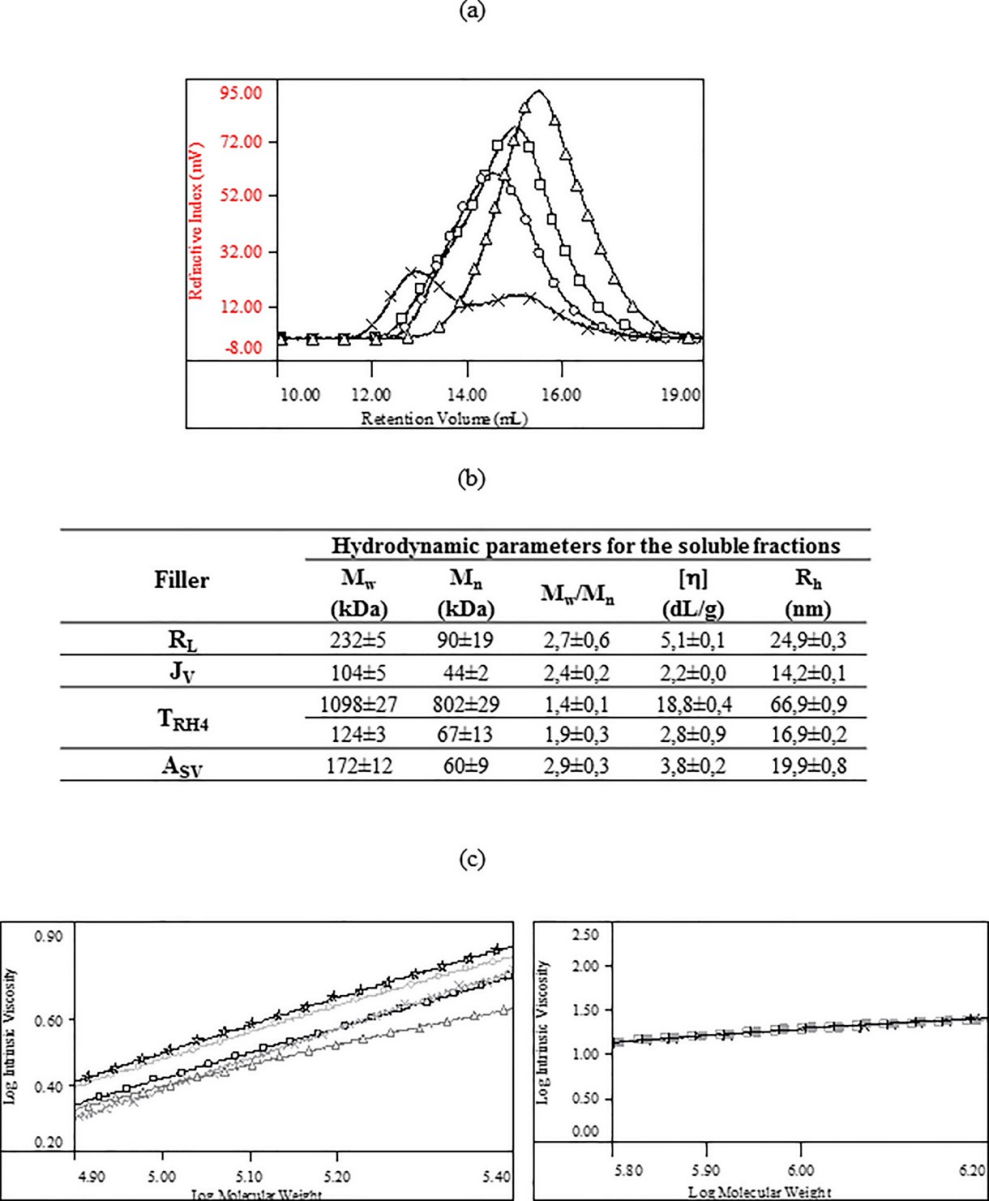
Results of the SEC-TDA characterization of the water-soluble fraction of the gels. (a) Overlap of the chromatographic profiles (RI signal): R_L_ (circles); J_V_ (triangles); T_RH4_ (×); A_SV_ (squares). (b) Complete chromatographic report: weight average molar mass (M_w_), numeric average molar mass (M_n_), polydispersity index (M_w_/M_n_), intrinsic viscosity ([η]), hydrodynamic radius (R_h_). For T_RH4_, the data resulting from the analysis of the two distributions are reported. The high molecular weight fraction represents the 53±1% w/w of the total soluble HA amount. (c) Overlap of representative Mark-Houwink-Sakurada curves for (on the left) a linear HA control (stars), R_L_ (circles), J_V_ (triangles), T_RH4_ II distribution (×) and A_SV_ (squares) and for (on the right) a linear HA control (empty and filled squares) and T_RH4_ I distribution (×).

The superimposition of the chromatographic profiles (RI signal) for the four samples and the related hydrodynamic parameters are reported in Figs 2A and 2B, respectively. The overlay in Fig 2A indicates the presence of one molecular size distribution for A_Sv_, R_L_ and J_V_ samples while two (partially overlapping) distributions can be clearly identified in the T_RH4_’s profile.

As shown in Fig 2B, the water-soluble HA in R_L_, J_V_ and A_Sv_ consists of low molecular weight chains (M_w_ in the range 100-230kDa) with similar distribution width (M_w_/M_n_: 2.4–2.9). The analysis of the two distributions of T_RH4_ revealed the presence of a low molecular weight fraction (about 120kDa M_w_) and of a high molecular weight fraction (about 1100kDa M_w_) with the latter representing 53±1% w/w of the total soluble HA amount.

The superimposition of the MHS curves obtained for the soluble fractions of the gels and for linear HA samples, available in our laboratories, is reported in Fig 2C. The comparison on the left shows that the low molecular weight soluble fractions of R_L_, J_V_, A_SV_ and of T_RH4_ (II distribution) exhibit lower intrinsic viscosity (more compact structure) than unmodified HA thus suggesting they are chains other than linear HA. The comparison on the right shows that the high molecular weight distribution of T_RH4_ (I distribution) exhibits a MHS curve that perfectly overlaps to the curve of linear HA samples indicating the same conformation for chains having the same length therefore supporting the presence of linear high molecular weight HA in T_RH4_ gel.

### Hydration capacity

**Fig 3:**
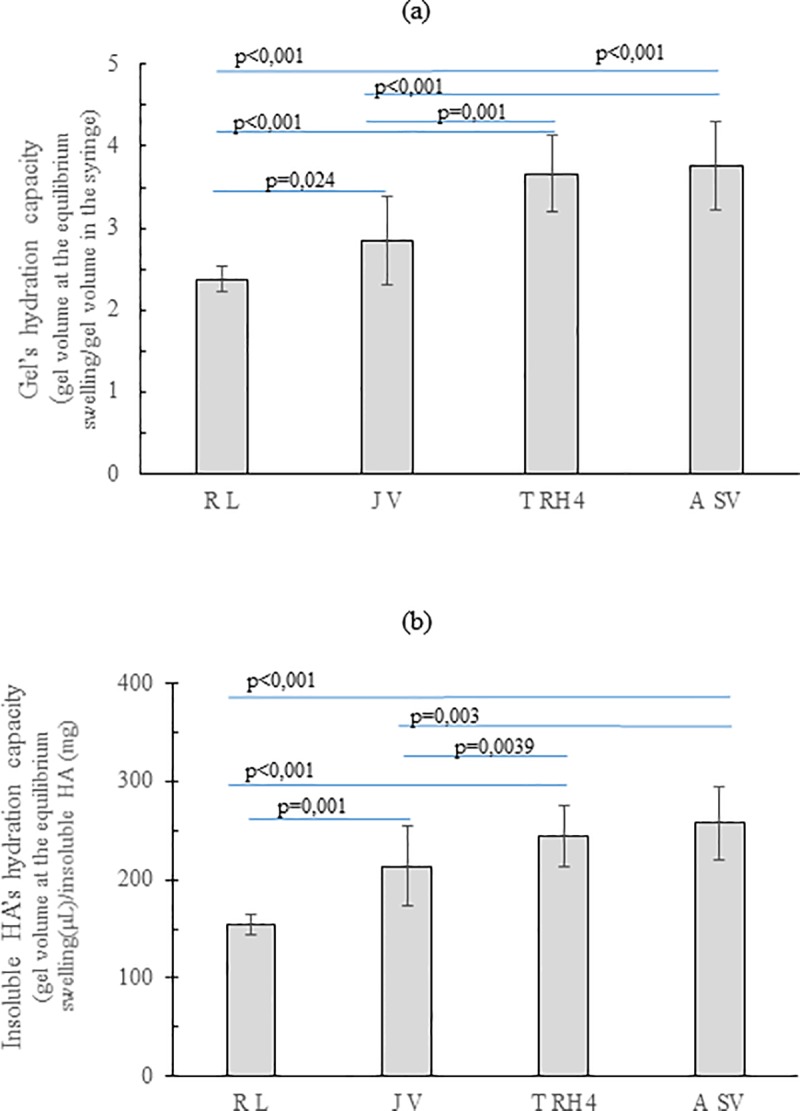
Filler hydration capacity in physiological medium. (a) Hydration capacity (volumetric expansion), at equilibrium, for the diverse gels. Data indicate the volume reached by 1mL of each gel when allowed to reach equilibrium in PBS. (b) Hydration capacity for the water-insoluble HA contained in each gel. Data indicate the volume reached, at equilibrium, by 1mg of water-insoluble HA.

The degree of the gel hydration in physiological medium is shown in Fig 3A. R_L_ showed about 2.4fold volume expansion making it the gel with the lowest hydration capacity. T_RH4_ and A_Sv_ expanded comparably (p >0.05) and exhibited the highest hydration capacity more than tripling their volume in the swollen state. J_V_ showed intermediate hydration extent.

The same data, normalized with respect to the insoluble HA amount (mg) in the gels, are reported in Fig 3B. The results show that the insoluble HA in T_RH4_ and A_SV_ uptakes similar water amounts (240–250 μL/mg; p >0.05) that are slightly higher than the ones entrapped by the insoluble HA in J_V_. The insoluble hydrogel in R_L_ proved the lowest swelling extent (about 150 μL/mg).

### Rheological parameters

**Fig 4:**
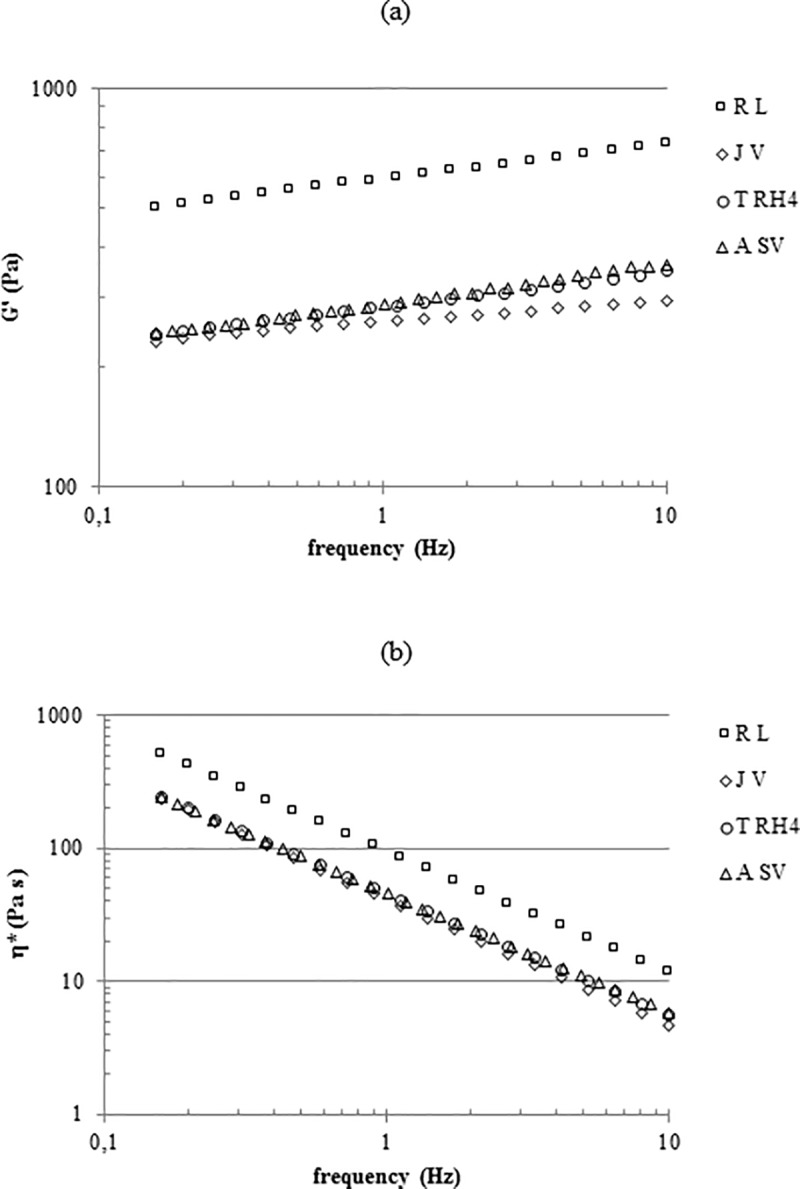
Rheological characterization. G’ values (a) and complex viscosity values (b) for the gels, as a function of the frequency. Measurements were performed at 37°C.

The rheological studies indicate that all the gels exhibit an elastic behavior with tan delta values in the range 0.10–0.15. As shown in Fig 4A, only a slight dependence of the Storage Modulus on the frequency was evidenced. T_RH4_, J_V_ and A_Sv_ were comparable for rigidity while R_L_’s stiffness was far higher: G’ values at 0.7Hz frequency are in the range 260–280 Pa for T_RH4_, J_V_ and A_SV_ and about 580Pa for R_L_. The complex viscosity profiles (Fig 4B) indicate a viscosity constantly decreasing with the frequency with A_SV_, J_V_ and T_RH4_ showing similar values in the whole range of frequencies and R_L_ proving the most viscous gel.

### Degradation studies

**Fig 5:**
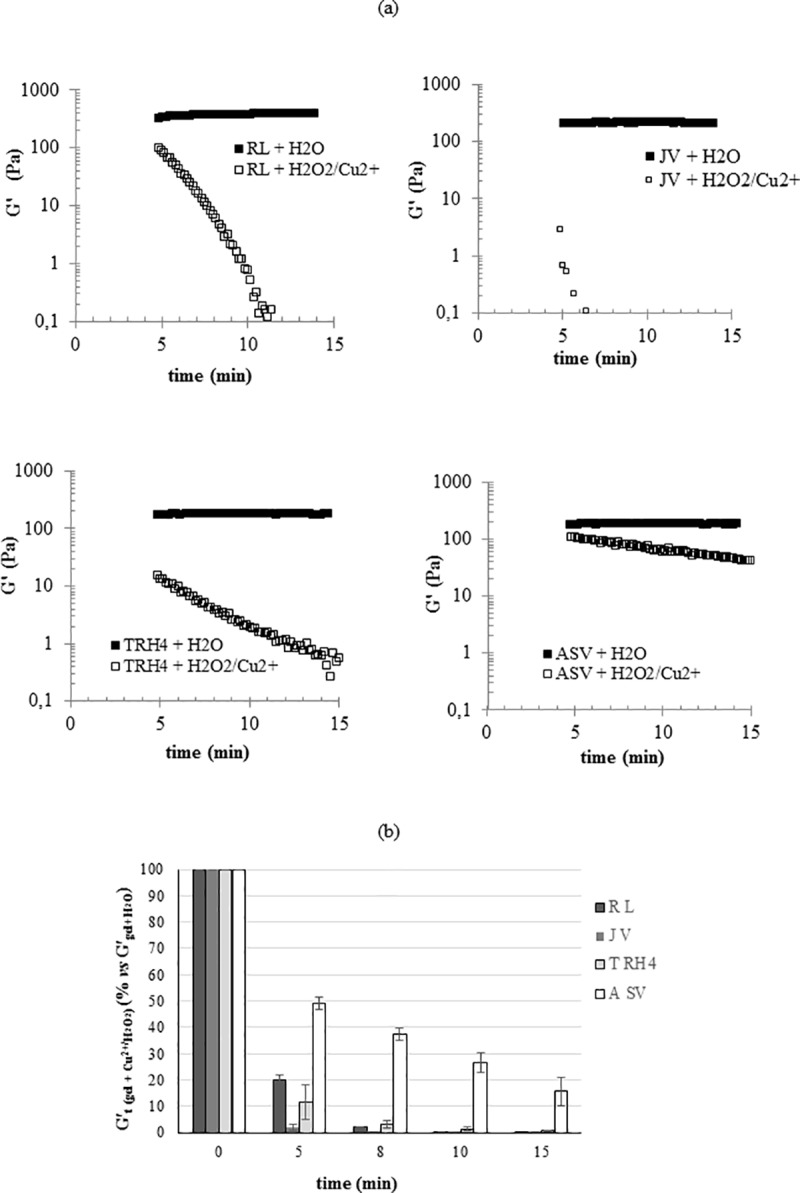
Degradation of the gels in the presence of the H_2_O_2_/Cu^2+^ system. (a) Reduction of G’ during 15 minutes of incubation with the degrading system. For each filler, the trend of the G’ value during incubation with water under the same conditions (control) is also reported. (b) Residual G’ (% compared to the control) at 5, 8, 10 and 15 minutes of incubation with the ROS generating system. Measurements were performed at 37°C.

The results of filler degradation in the presence of the H_2_O_2_/Cu^2+^ system are reported in Fig 5. In particular, the G’ values registered during filler incubation with the ROS generating system in comparison to the G’ values registered for the control (gels incubated with water) are reported in Fig 5A. As shown, after dilution with water, 1.1–1.4fold lower G’ values were registered for the gels and such values remained constant during the measurements. After the same dilution with the ROS generating system, a drop in G’ was observed for all the gels showing they are sensitive to degradation under the applied conditions. By comparing the profiles of the fillers, it becomes clear that while for R_L_, A_SV_ and T_RH4_ a progressive reduction in G’ could be observed in the measuring time interval, J_V_’s degradation occurred faster and, at the beginning of the measurement, rigidity had already reached values close to the minimum measurable ones, thus causing a scattered signal. The data, normalized with respect to the controls are reported in Fig 5B. In particular, the residual G’ values (% with respect to the value in the absence of the degrading system) after 5, 8, 10 and 15 minutes of incubation are reported. The data clearly show different rates of G’ reduction with A_Sv_ and J_V_ proving the most resistant and the most sensitive gel, respectively. Specifically, J_V_ was totally degraded (98% loss in G’) at only 5 minutes of incubation while A_SV_ still preserved 50% of its G’ value at the same time of incubation with a final G’ value 6.4fold lower than the initial one. R_L_ and T_RH4_ showed an intermediate stability and both were completely degraded at 10 minutes of incubation (more than 99 and 98% loss in G’, respectively).

The degradation studies carried out in the presence of BTH demonstrated that, under the applied conditions (4h of incubation with BTH 100U/mL), all the gels completely solubilized (water-soluble HA fraction higher than 96wt%).

### Cohesivity

**Fig 6:**
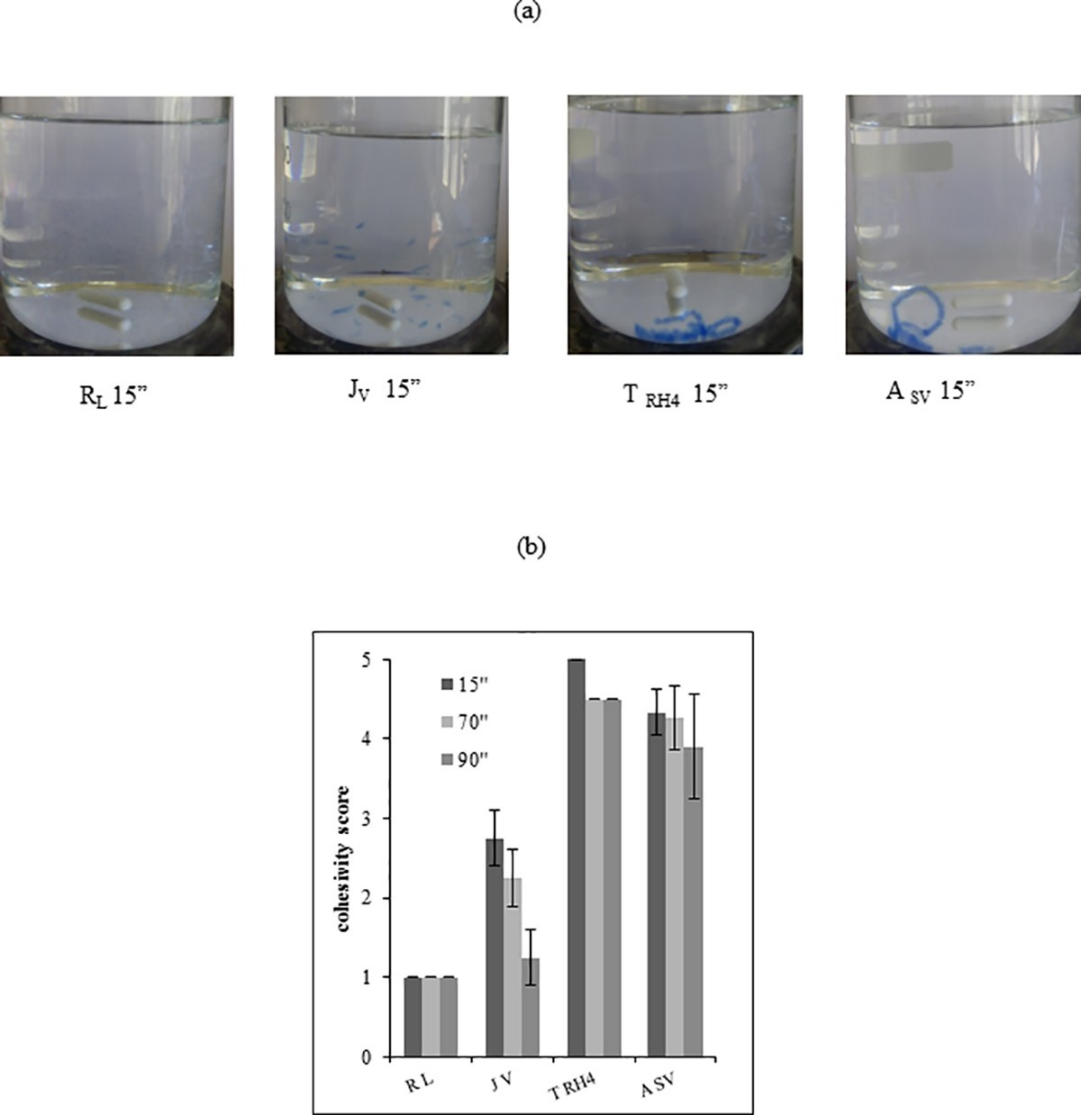
Gel cohesivity. **(a)** Images of the gels captured at 15 seconds after starting the test. (b) Cohesivity score assigned to the gels at diverse time intervals according to the Gavard-Sundaram Cohesivity Scale [33].

The results from the cohesivity test are reported in Fig 6. Images in Fig 6A, captured at 15” after the starting of the test, clearly show the fillers greatly differ in behavior. Upon contact with water, both J_V_ and R_L_ broke up into pieces with R_L_ giving rise to very small particles highly dispersed into water. In contrast, T_RH4_ and A_SV_ exhibited a highly cohesive behavior even if a slight fragmentation was observed for A_SV_. The cohesivity score assigned to the gels all over the experiment, according to the Gavard-Sundaram Cohesivity Scale [33], is reported in Fig 6B. No further significant changes were observed for A_SV_ and R_L_ in the interval time of observation while J_V_ progressively disaggregated reducing its cohesivity score down to the same value as R_L_. Fragmentation, at very low extent, was observed also for T_RH4_. Overall, A_SV_ and T_HR4_ nearly completely retained their *structur*e. It is worth citing that R_L_ and J_V_ still presented visible suspended particles at time intervals far longer than 90” while A_SV_ and T_RH4_ progressively lose definition however no visible particles could be detected and the samples appeared as uniformly colored solutions.

### Biological studies

HDF after 48h culture in the presence of the fillers were observed at the optical microscope and compared to untreated cells (ctr). Cells exhibited typical morphology and even the cell number was found not significantly varied in respect to the control, regardless of the specific treatment (representative images are shown as Supplementary material, S1 Fig).

**Fig 7:**
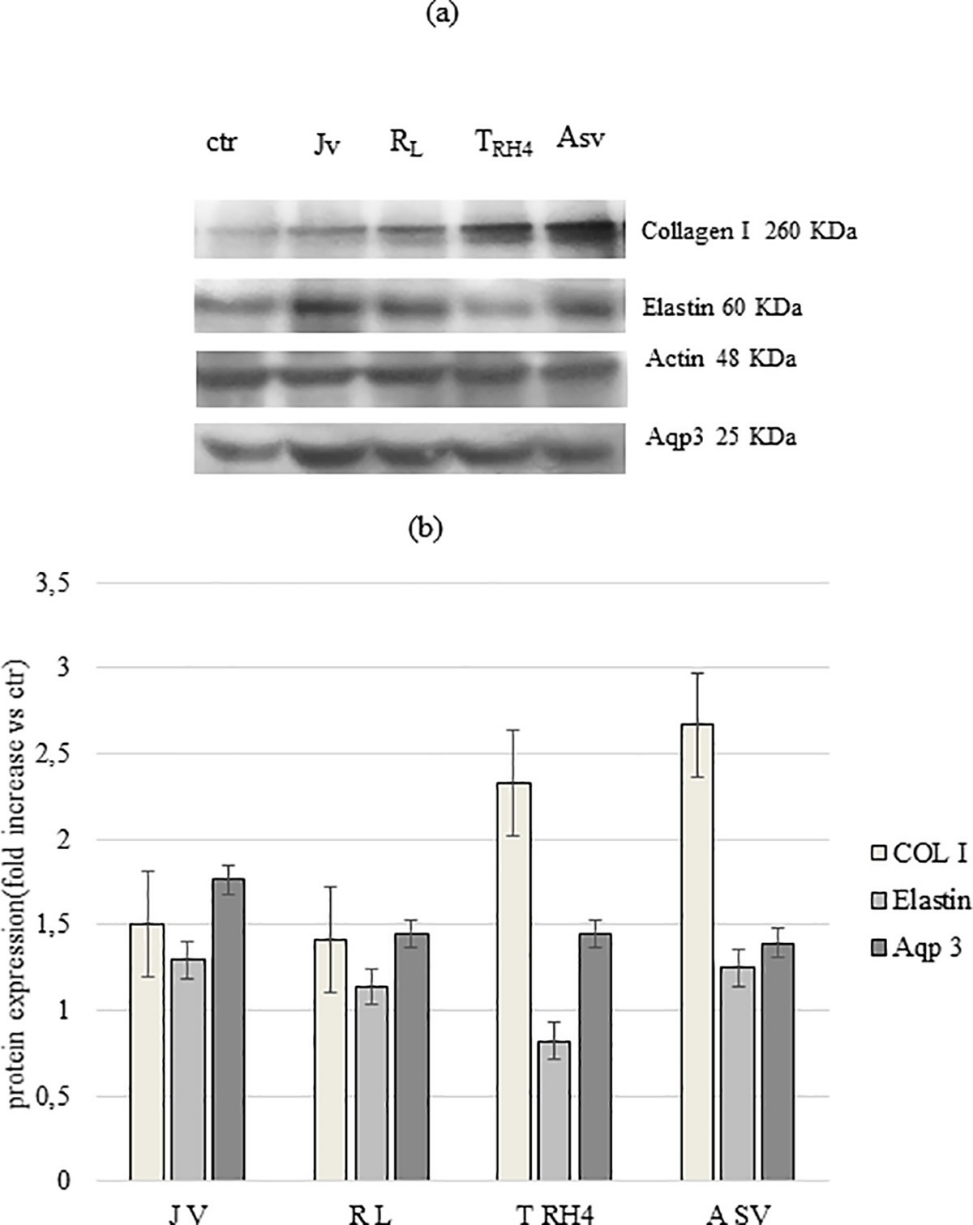
Western blotting analyses. Western blotting image (a) and densitometric analysis (b) of Collagen I, Elastin and Aqp3 expression in HDF after 48h of incubation with the gels. Actin was used to normalize the results. Protein expression is reported as fold increase compared to ctr (untreated cells).

The results of the protein expression analyses are reported in Fig 7. Specifically, a western blotting image is reported in Fig 7A. The quantitative expression of the investigated proteins, normalized to untreated cells (ctr), is reported in Fig 7B. Collagen type 1 expression level was up-regulated by all the fillers tested with the highest increase in expression registered for A_SV_ and T_RH4_. With regard to Aqp3, as for Collagen Type I, the protein levels were enhanced by all the treatments. However, in this case, J_V_ was responsible for the highest level of protein expression (about 2-fold in respect to control). Slighter or no up-regulation was found for elastin.

## Discussion

To provide biophysical and biochemical parameters describing HA hydrogels clinically used as volumetric dermal fillers, an extensive *in vitro* characterization of four commercial gels was carried out. New methods to evaluate this kind of hydrogels were also presented.

The content in water soluble/insoluble HA was the first feature investigated. It is well known that the HA concentration, declared for filler formulations, refers to both the insoluble HA hydrogel and to an additional water-soluble fraction of the biopolymer that is generally added to facilitate product extrusion through a needle of proper dimension (27-30G) [18, 21, 37]. The analyses (Fig 1) revealed that the latest generation of fillers tested are formulated with a high amount of water soluble polymer. Such amount spans from 23 up to about 40% w/w of the total biopolymer content and is about 2-4fold higher than the values mostly reported to date [18, 21].

Further, most of the gels tested are equivalent in amount of water-insoluble HA and differ only for the soluble HA content. Considering that the soluble fraction of the biopolymer is not responsible for all of the performance (i.e., lifting capacity, stability to degradation), the listed HA concentration in commercial fillers may not directly correlate to the final gel behavior [23].

Based on the hydrodynamic analyses (Fig 2), the soluble HA fraction of the volumetric fillers consists of chemically modified short (M_w_ < 250kDa) HA chains that can reasonably derive from the manufacturing and/or the final sterilization that may release a certain amount of chemically modified, but not “insolubilized” HA. The molecular weight of HA undergoing crosslinking could also affect the extent of soluble HA in the final gel. However, one of the tested gels (T_RH4_), unlike the others, contains a relevant fraction of unmodified high molecular weight HA that was most probably added to the formulation.

One of the main crucial features of a dermal filler is its hydration capacity, representing the amount of water that it can retain. This capacity is responsible, *in vivo*, for the restoration of a high level of tissue hydration contributing to volume augmentation. The class of volumetric gels tested here proved a hydration extent (Fig 3A) comparable to the values reported for high-volumizing products [21] and, among the gels, R_L_ stands out for its lesser water absorption ability. It is worth underlying that the data reported (Fig 3A) do not directly translate into the fillers relative *in vivo* expansion after injection: for a more accurate prediction, the capacity of the gels to counteract the compression forces of the surrounding tissues has to be taken into account.

Swelling properties are due to the insoluble hydrogel, therefore the swelling extent normalized with respect to the insoluble HA content was calculated (Fig 3B). These data are related to the structure of the HA network. Generally, the greater the crosslinking within a hydrogel, the lower its swelling capacity. Based on this, data suggest a higher crosslinking extent for R_L_ hydrogel while A_SV_, J_V_ and T_RH4_ exhibit similar crosslinking density even if manufactured according to diverse technologies.

The projection capacity is the main characteristic for a “volumetric” filler and is related to the gel’s rheological properties, especially to rigidity. Compared to the literature, the gels tested here exhibited high G’ values (Fig 4A) that are in agreement with already reported data and consistent with their volumizing indications and suggested application site [22]. While J_V_, T_RH4_ and A_SV_ presented similar G’ values (200-300Pa) in the physiologically relevant frequency range, R_L_ stands out with far higher rigidity thus suggesting, for this filler, the highest projection capacity. This would be in line with the greater crosslinking predictable for this gel based on the relative hydration. Fillers ranking in terms of complex viscosity resembles, as expected, the one in rigidity (Fig 4B). Besides affecting ease of delivery, viscosity, along with cohesivity, is supposed to play a key role in tissue integration pattern [3, 22]. Cohesivity, a recently investigated parameter, is conceived as a measure of the integrity maintenance of the gel in physiological conditions. The better a gel can spread without disaggregating (low viscosity and high cohesivity), the better its tissue integration, therefore the more natural the aesthetic outcome. Based on this, the relative rank in cohesivity and viscosity would suggest J_V_ and A_SV_ as the gels with the better tissue integration pattern while a poorer performance could be predicted for R_L_. Nevertheless, it was surprising that although the gels tested had similar indications, they greatly differ in cohesivity. The manufacturing parameters directly related to this property have still to be fully identified. However, it is noteworthy that the data obtained here are in agreement with a correlation previously suggested between swelling /rigidity and cohesive properties: the higher the water uptake ability and the lower the G’ value, the higher the cohesivity of the gel [22].

HA filler degradation profile is usually widely investigated since it is related to the clinical effect longevity [38]. Even if *in vivo* degradation is mainly due to the action of both hyaluronidases and ROS, the main factor studied up to now is gel sensitivity to BTH-catalyzed hydrolysis [18, 21, 39–41] with a very few studies reporting on commercial filler stability to ROS species [41–42]. Here, the complete solubilization of the gels due to enzymatic hydrolysis was demonstrated. This indicates that the HA networks are still recognized by the enzyme thus ensuring products bioresorbability. These data, along with the sound viability and proliferation found for HDF cultured in the presence of fillers in the medium (0.16% w/w final concentration), confirm the biocompatibility of the chemically modified HA samples.

Further, a model of *in vitro* oxidative stress was employed to study the rate of depolymerization of the volumetric fillers induced by reactive oxygen species (ROS). The composition of the ROS generating system was successfully set to have significant degradation for all the samples in the timeframe of the experiment and to appreciate the diverse gel sensitivity. Based on the collected data, A_SV_ and J_V_ can be predicted as the longer and the shorter lasting gels, respectively. It would be of interest to unravel the parameters affecting filler behavior in the presence of ROS. Here, the sensitivity to ROS seems to correlate to both the degree of crosslinking and the gel cohesivity with a degradation rate decreasing at the increase of both parameters. In particular, among the gels with similar predictable crosslinking, the most cohesive were also the most stable gels with A_SV_ exhibiting far higher stability (maybe correlated to the higher biopolymer concentration and/or extent of chemical modification). Even if the lowest in terms of cohesivity, R_L_ exhibited stability comparable to T_RH4_ may be due to its higher crosslinking density requiring longer for rigidity to be lost.

Lately, interest in demonstrating the biological effect of fillers beyond the volumizing action, has been growing. This effect could explain the fact that after filler injection, the patients often report improved skin quality lasting longer than the filler average half-life let us expect [43, 44]. The cellular *in vitro* model applied here provides interesting data in this direction. In our HDF cultures, HA volumetric fillers boosted the expression of connective extracellular matrix components, such as Collagen type I and elastin, and also of AQP3. The latter is an abundant skin aquaglyceroporin, a water channel that plays an important role in the hydration of mammalian skin epidermis. The data suggest an efficient effect of commercial fillers on tissue structure mechanical remodeling. A positive effect on skin elasticity, tone and hydration can be finally predicted. Such results corroborate previous *in vivo* and *in vitro* studies reporting on the cosmetic effects of HA-based dermal fillers, associated with their ability to stimulate collagen synthesis via induction of fibroblasts, finally improving skin quality [21, 43–48].

To date, the most accredited hypothesis about HDF activation by HA fillers is a mechanical stimulation [49]. It is opinion of the authors that the soluble HA fractions of dermal fillers could also play a role. This would be consistent with the better high performance of A_SV_ and T_RH4_ in fostering COL I production. However, the higher effect of J_V_ on Aqp3 synthesis should be differently explained. Further studies are needed to better evaluate this issue. Considering the effect reported for naturally occurring HA on skin remodeling, the molecular weight and the extent of modification of both the water-soluble HA chains of the fillers, and of the polymeric chains between two crosslinking points (in the insoluble hydrogel), would influence final biological effect. These parameters are expected to be responsible, at a certain extent, also for the diverse physical properties of the gels.

## Conclusions

A broad *in vitro* characterization of HA-based dermal fillers with the same indications as volumetric gels was accomplished. Data allowed us to establish a range of rheological properties, hydration capacity, HA soluble fraction extent and cohesivity that well characterize these products. Differences in behavior among the fillers were also highlighted. Further, degradation due to ROS was systematically monitored exploiting rheological measurements proving diverse sensitivity of the gels tested. Finally, the boost in elastin, collagen and Aqp3 production for HDF cultured in the presence of the fillers was demonstrated thus supporting the lately emerged opinion of a biological positive effect of HA dermal fillers on skin restoration and hydration.

## Supporting information

S1 Fig Micrographs at the optical microscope of HDF after 48h of incubation with the gels (0.16% w/w in the culture medium) and of untreated cells (ctr). (TIF)
