# Supplementary figures and images for: Hyaluronan-based hydrogels as dermal fillers: The biophysical properties that translate into a “volumetric” effect
Source: PLoS One. 2019 Jun 11;14(6):e0218287. doi: 10.1371/journal.pone.0218287 (PMC6559669; doi:10.1371/journal.pone.0218287)

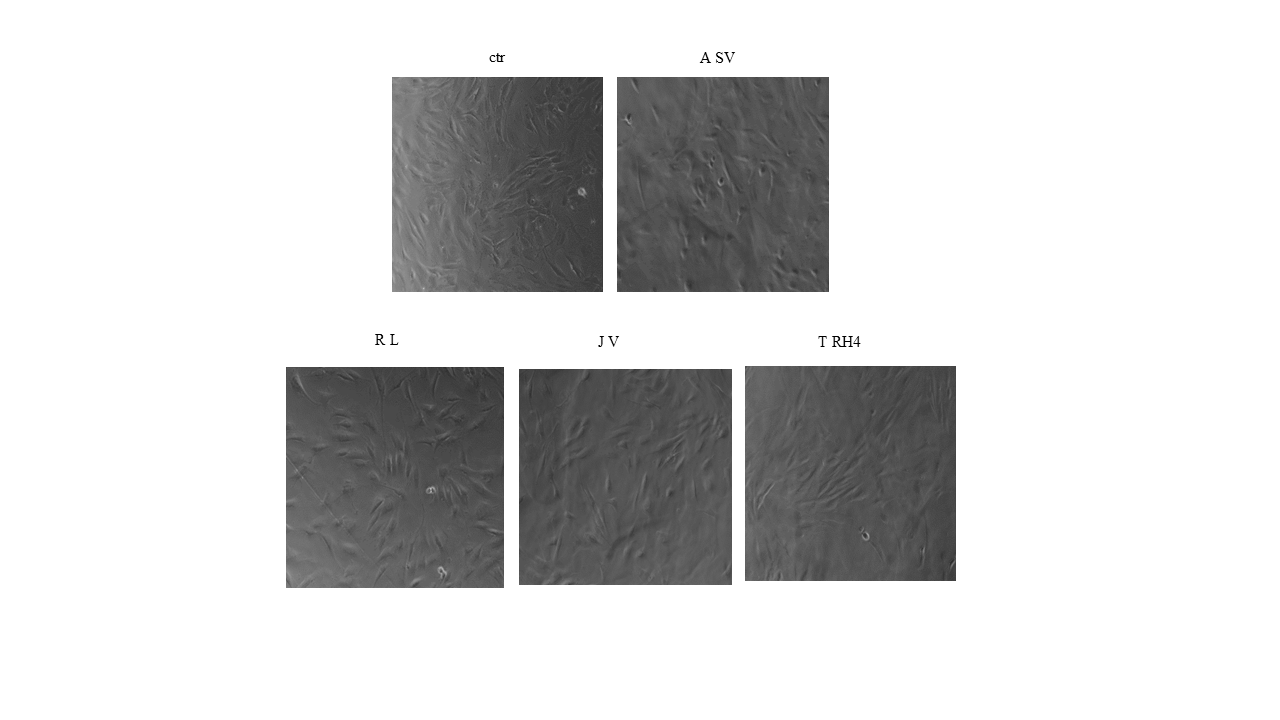

Supplement: S1 Fig — (TIF) [file pone.0218287.s001.tif]
